# Scavenger receptor A1 participates in uptake of *Leptospira interrogans* serovar Autumnalis strain 56606v and inflammation in mouse macrophages

**DOI:** 10.1080/22221751.2021.1925160

**Published:** 2021-05-18

**Authors:** Yanchun Wang, Xia Fan, Lin Du, Boyu Liu, Haihan Xiao, Yan Zhang, Yunqiang Wu, Fuli Liu, Yung-Fu Chang, Xiaokui Guo, Ping He

**Affiliations:** a Department of Medical Microbiology and Parasitology, Shanghai Jiao Tong University School of Medicine, Shanghai, People’s Republic of China; b Department of Clinical Laboratory, Fudan University Shanghai Cancer Center, Shanghai, People’s Republic of China; c College of Veterinary Medicine, Cornell University, Ithaca, NY, USA; d Key Laboratory of Parasite and Vector Biology, Ministry of Health; School of Global Health, Chinese Center for Tropical Diseases Research, Shanghai Jiao Tong University School of Medicine, Shanghai, People’s Republic of China

**Keywords:** *Leptospira interrogans*, macrophage, scavenger receptor A1, phagocytosis, inflammatory response

## Abstract

Leptospirosis, caused by pathogenic *Leptospira* species, has emerged as a widespread zoonotic disease worldwide. Macrophages mediate the elimination of pathogens through phagocytosis and cytokine production. Scavenger receptor A1 (SR-A1), one of the critical receptors mediating this process, plays a complicated role in innate immunity. However, the role of SR-A1 in the immune response against pathogenic *Leptospira* invasion is unknown. In the present study, we found that SR-A1 is an important nonopsonic phagocytic receptor on murine macrophages for *Leptospira*. However, intraperitoneal injection of leptospires into WT mice presented with more apparent jaundice, subcutaneous hemorrhaging, and higher bacteria burdens in blood and tissues than that of SR-A1^-/-^ mice. Exacerbated cytokine and inflammatory mediator levels were also observed in WT mice and higher recruited macrophages in the liver than those of SR-A1^-/-^ mice. Our findings collectively reveal that although beneficial in the uptake of *Leptospira* by macrophage, SR-A1 might be exploited by *Leptospira* to modulate inflammatory activation and increase the susceptibility of infection in the host. These results provide our new insights into the innate immune response during early infection by *L. interrogans*.

## Introduction

Leptospirosis, known as Weil’s disease, is caused by pathogenic species of the genus *Leptospira*, which has emerged as the most widespread zoonotic disease worldwide [1,2]. Human leptospirosis is an acute febrile illness with a wide range of clinical features from mild flu-like symptoms to severe leptospirosis characterized by jaundice, bleeding, pulmonary hemorrhage, renal failure and death [3].

Innate immune responses constitute the first line of defense against *Leptospira*. At the early stages of *Leptospira* infection, macrophages play a complicated role against *Leptospira* by phagocytosis and induction of signalling pathways to produce pro-inflammatory cytokines and antigen presentation [4–6].

The process of bacterial recognition and phagocytosis by macrophages has been intensively studied in recent decades [7,8]. A comprehensive series of surface receptors have been identified as being involved in the phagocytosis of microorganisms, including integrins, Fc gamma receptors (FcγRs), mannose receptors, and scavenger receptors, etc. [9]. In the case of *Leptospira*, our understanding of the receptors involved in phagocytosis is limited. Two phagocytic receptors described for *Leptospira* are the third complement receptor (CR3) and β2 integrin, and much less is known whether other receptors are involved in leptospiral phagocytosis [10,11].

Scavenger receptor A1 (SR-A1), also called macrophage scavenger receptor (MSR) or CD204, belongs to a class of pattern recognition receptors (PRRs) expressed primarily on macrophages [12]. SR-A1 was initially described as a receptor for modified lipoproteins involved in atherosclerosis development [13]. It has been identified as being involved in many critical biological processes, such as adhesion and phagocytosis [14]. Studies have shown that SR-A1, as a nonopsonic phagocytic receptor, can bind and phagocytose various bacteria, such as *Escherichia coli*, *Neisseria meningitidis*, *Streptococcus pneumoniae*, *Staphylococcus aureus* and *Listeria monocytogenes* [15–18]. In addition, there is evidence that SR-A1 is involved in regulating innate immune responses and proinflammatory cytokine responses to pathogen infection [19]. However, no studies have been reported about the interactions of SR-A1 with *Leptospira*. Thus, we sought to evaluate the role of SR-A1 during the *Leptospira* infection.

Animal models represent essential tools in research on the pathogenic mechanism of leptospirosis. Guinea pigs and hamsters have been the most commonly used animal models for *Leptospira* infection [20]. We recently developed a murine model of acute and self-resolving leptospirosis by infecting adult, immunocompetent C57BL/6 mice with *L. interrogans* serovar Autumnalis strain 56606v [21]. This murine leptospirosis model closely recapitulates natural disease in humans, with characteristic manifestations including prominent jaundice and pulmonary hemorrhage [21]. Thus, we use WT and SR-A1-/- mice to investigate the host–pathogen interactions of leptospirosis.

Our results showed that SR-A1 is an important nonopsonic phagocytic receptor for *Leptospira* ingestion. Additionally, SR-A1 contributes to regulating proinflammatory cytokine responses to *Leptospira* infection and affects the disease outcome. These results provide our new insights into the innate immune response during early infection by *L. interrogans*.

## Materials and methods

### Bacterial strains

Pathogenic *L. interrogans* serovar Autumnalis strain 56606v was kindly provided by the Institute for Infectious Disease Control and Prevention (Beijing, China), and serial passages in guinea pigs to maintain bacterial virulence. Leptospires *in vitro* were cultivated in EMJH medium at 28 °C to mid-log phase. After counting in a Petroff-Hauser chamber under dark-field microscopy, *L. interrogans* were suspended in PBS at a particular cell density for the experiment. The bacterial suspensions were then fixed for 1 h at 4°C with 4% paraformaldehyde to inactivate the bacteria. These preparations were washed and resuspended in PBS for use.

### Cell culture

Murine bone marrow-derived macrophages (BMDMs) were generated from WT and SR-A1^-/-^ mice, as previously described with minor modifications [22]. Briefly, tibiae and femurs from mice were flushed with PBS, and bone marrow cells were resuspended in RPMI 1640 (Gibco, USA) supplemented with 10% heat-inactivated FBS, 30% L929-conditioned medium, 1% HEPES and 1% Penicillin–Streptomycin (Gibco, USA). Cells were cultured in 10 cm Petri dishes (Nunc, Denmark) for 5 days at 37 °C in a humidified incubator with 5% CO_2_. Then, macrophages were obtained by scratching in cold PBS containing 0.5% EDTA, followed by centrifugation at 300×*g* for 10 min. The cells were washed again and subsequently cultured for experimental use.

Peritoneal macrophages (PMs) were harvested from WT and SR-A1^-/-^ mice after injection of 1 mL of 5% thioglycollate broth intraperitoneally, as previously described [23]. After lavaging the peritoneal cavity with 10 mL of cold RPMI 1640 followed by centrifugation at 300×*g* for 10 min, the cells were subsequently cultured in Petri dishes of 10% FBS RPMI 1640 for 2 h allowing macrophages to adhere.

RAW264.7 and HEK293 T cells, originally from American Type Culture Collection, were cultured in DMEM (Corning, USA) containing 10% FBS and 1% Penicillin–Streptomycin maintained at 37 °C in a humidified 5% CO_2_ incubator.

### Plasmid construction and transfection

Full-length murine macrophage SR-A1 cDNA ORF was cloned into a pHBLV-U6-ZsGreen-Puro plasmid (Hanbio, China) for lentiviral production. The recombinant retrovirus was transfected into RAW264.7 and HEK293 T cells according to the manufacturer’s instructions. Stable cells were screened with 800 μg/mL puromycin. Efficiency expression of SR-A1 was determined by the Western blotting and flow cytometry (FCM) method.

### Leptospires stimulation and phagocytosis

Cells seeded at 2 × 10^5^ per well were incubated on slides in a 24-well plate under appropriate culture conditions. Cells were infected with *L. interrogans* strain 56606v at MOI of 10–100 paraformaldehyde-fixed bacteria or 100 live bacteria per cell, respectively. To synchronize the stage of infection, the plates were centrifuged at 100×*g* for 10 min and then co-incubated in a humidified 5% CO_2_ incubator for 1 h. The cells were washed extensively with pre-cooled PBS to stop phagocytosis and remove extracellular *L. interrogans*.

In phagocytosis experiments, cells were pre-incubated before leptospires infection with polyI (100 μg/mL, Sigma, USA), polyC (100 μg/mL, Sigma), SR-A1 monoclonal antibody 2F8 (30 μg/mL, AbD serotec, United Kingdom), isotype control rat IgG2b (30 μg/mL, AbD serotec), cytochalasin D (20 μM, Sigma), mannan from *Saccharomyces cerevisiae* (10 mg/mL, Sigma) or N-Acetyl-D-glucosamine (50 μM, Sigma) for 30 min in the condition without FBS. Rabbit anti-*L. interrogans* strain 56606v antibody was used as the specific primary antibody. FITC-conjugated (BD, USA) or Alexa Flour 647-conjugated anti-rabbit IgG (Abcam, USA) as a secondary antibody was used before permeabilization (1% paraformaldehyde and 0.5% Triton X-100), while TRITC-conjugated anti-rabbit IgG (ProteinTech, USA) was used after that. Nuclei were stained with 1 μM DAPI for 10 min, and slides were sealed and detected by laser using an Olympus Confocal microscopy.

Thus, phagocytic leptospires inside macrophages were only stained by one fluorescein-labelled antibody after permeabilization, while diverse conjugated antibodies stained leptospires adhering outside both pre- and post- permeabilization. Two hundred macrophages were counted in several HP fields, and the rates of macrophages with phagocytosed leptospires were calculated.

### Experimental Animals

SR-A1-deficient (SR-A1^-/-^) mice on the C57BL/6 background were kindly provided by Prof. Qi Chen, Nanjing Medical University [24]. Age (8 weeks old) and gender (female)-matched WT C57BL/6 mice obtained from Shanghai Jiao Tong University School of Medicine were used as controls. Primary cells from BALB/c mice were used in the experiment of SR-A1 inhibition because the commercial antibody 2F8 did not block the SR-A1 of C57BL/6 mice [25]. These mice were maintained under specific-pathogen-free conditions in the vivarium of the Experimental Animal Center at Shanghai Jiao Tong University School of Medicine. All experiments were performed in strict accordance with the Regulations for the Administration of Affairs Concerning Experimental Animals. The Animal Ethics Review Committee of Shanghai Jiao Tong University approved all animal procedures (project number A-2018-021).

### *Leptospira* infection with WT and SR-A1^-/-^ mice

WT and SR-A1^-/-^ mice were infected with 2 × 10^8^ leptospires in 200 μL PBS via the intraperitoneal (IP) route according to the method described [26]. Negative control mice were intraperitoneally injected with 200 μL PBS. The mice were bled and sacrificed at 1, 2, 3, and 5 days post-infection (dpi), respectively. The blood, liver and kidney of WT and SR-A1^-/-^ mice were collected for RNA extraction, histological and immunohistochemical analysis.

For the test of phagocytosis of PMs to leptospires *in vivo*, the mice were sacrificed at 2 and 24 h post-infection (hpi), respectively. PMs were harvested and cultured on slides in a 24-well plate. Adherent or phagocytized leptospires were labelled and detected by specific antibodies as the method mentioned above.

### Bacterial loads

The burden of leptospires in murine blood or tissues was analysed by reverse transcription and real-time PCR according to the method described [21]. The concentration of leptospires in the animal blood and tissues was quantified with an ABI 7500 PCR System (Applied Biosystems) using Power SYBR Green PCR Master Mix (Applied Biosystems). The concentration of the final PCR product (16S rRNA: 5’-AGC ACG TGT GTT GCC CTA GAC ATA-3’ and 5’ -GTT GCC ATC ATT CAG TTG GGC ACT-3’) was calculated by 2^−ΔCt^ relative to GAPDH as the reference gene.

### Pathological and immunohistochemical studies

Tissues (liver and kidney) were collected and fixed in 4% paraformaldehyde and then embedded in paraffin. Tissue sections were stained with hematoxylin and eosin (H&E). Tissue injury was examined by light microscopy. Immunohistochemical staining, using *L. interrogans* strain 56606v-specific rabbit antiserum prepared in our lab, was performed using the EnVison system (Dako, Glostrup, Denmark).

### Serum biochemical analysis

The serum was collected from blood samples and stored at -80 °C. The concentrations of total bilirubin (TBIL), aspartate transaminase (AST) and serum creatinine (CREA) were measured by using a UniCel DxC 800 Synchron autoanalyzer (Beckman Coulter, Brea, CA, USA).

### Quantification of surface receptors, inflammatory mediator and cytokines in macrophages and tissues

WT and SR-A1^-/-^ PMs were infected by alive *L. interrogans* strain 56606v as MOI = 100 for 2 h *in vitro*, and then 100 μg/mL gentamicin was used for 1 h to kill the extracellular leptospires. Culture medium was replaced and primary PMs were extracted for RNA detection (marked as 3 hpi). For protein detection, PMs were kept on culture for 1 d (1 dpi) and supernatants or lysates were collected.

TRIzol LS Reagent (Thermo Scientific, USA) was used for RNA extraction from primary cells stimulated by leptospires *in vitro*, and from blood or tissues infected by leptospires *in vivo*. Reverse transcription was used by a SuperScript III First-Strand Synthesis SuperMix Kit (Thermo Scientific), and cDNA was then subjected to real-time quantitative PCR (qPCR) with ABI 7500 PCR System (Applied Biosystems, USA). Primers were listed in the supplemental material (Supplementary Table 1). Real-time PCR data were calculated by 2^−ΔΔCt^ relative to GAPDH as the reference gene and cells without leptospiral stimulation as control.

The cytokine detection of supernatants was performed using ELISA kits (R&D Systems, USA) for mouse IL-1β, IL-6 and TNFα, according to the instructions provided by the supplier. Nitric oxide (NO) formation was quantified immediately via the Griess reaction.

For caspase-1 and IL-1β detection by Western blot analysis, cultured PMs supernatants and lysates were treated by methanol and chloroform with vortex and centrifugation at 12000 rpm × 5 min. Total proteins extracted from supernatants and lysates of PMs were run on 12% SDS-PAGE and then blotted onto polyvinylidene fluoride (PVDF) membranes (Millipore, USA) using the Bio-RAD Trans-Blot Turbo^TM^ transfer system. Membranes were incubated for 1 h in 5% non-fat milk diluted in Tri-buffered saline, 0.1% Tween20 (TBST). Mouse anti-caspase-1 monoclonal antibody (1:1000) (Adipogen, USA) and Rabbit anti-IL-1β monoclonal antibody (1:1000) (CST, USA) were applied as primary antibodies. The mouse anti-caspase-1 or the rabbit anti-IL-1β was added first and incubated overnight at 4 °C. After 4 washing with TBST, HRP-conjugated antibodies (1:2000) (Beyotime, China) were applied as secondary antibodies and incubated at room temperatures for 1 h. After 4 washes, the membranes were visualized using a chemiluminescent substrate (Thermo Scientific, USA) and exposed to ImageQuant LAS4000 (GE Healthcare, USA).

### Statistical analysis

Statistical analysis was performed using SPSS version 20.0 (IBM Corporation, USA), and figures were generated using GraphPad Prism software version 5.0. The data were expressed as means ± SEM. The mean values obtained from the experiments were compared utilizing Student’s *t*-test, one-way ANOVA analysis with post-hoc test or multiple *t*-tests. A *P*-value of less than 0.05 was considered significant.

## Results

### SR-A1 is an important receptor for nonopsonic-phagocytosis of *L. interrogans* by murine macrophage

**Figure 1.**
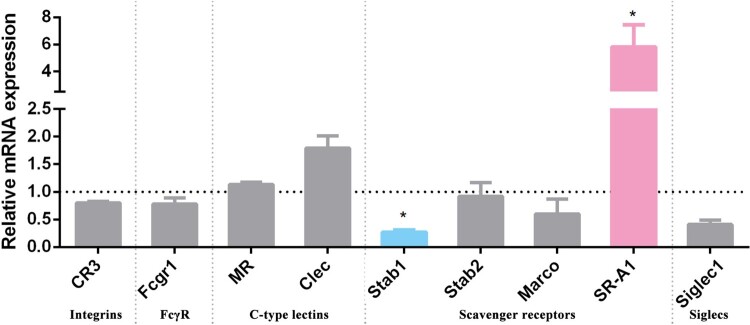
Regulation of surface receptors of PMs by stimulation with *L. interrogans* strain 56606v. PMs were incubated with *L. interrogans* strain 56606v for 3 h. Total RNA was extracted and reverse transcribed cDNA was detected by qPCR method. Relative expression of surface receptors was calculated by 2^−ΔΔCt^ relative to GAPDH as the reference gene and cells without leptospiral stimulation as control. These data were expressed as the mean ± SEM from at least three experiments. *P < 0.05 and significant in multiple *t*-tests.

We assessed the regulation of candidate macrophage surface receptors in response to stimulation with *L. interrogans* 56606v in order to screen the receptors participating in the recognition of *Leptospira*. Integrins, Fc receptors, C-type lectins, and sialic acid-binding Ig-like lectins (siglecs), and several other receptors belonging to the scavenger receptor family were regulated by stimulation with *L. interrogans* (Figure 1). Notably, only the expression of SR-A1 from these candidate receptors was found to be significantly increased, further suggesting the potential role of SR-A1 in the interaction of macrophages and *Leptospira*.

**Figure 2.**
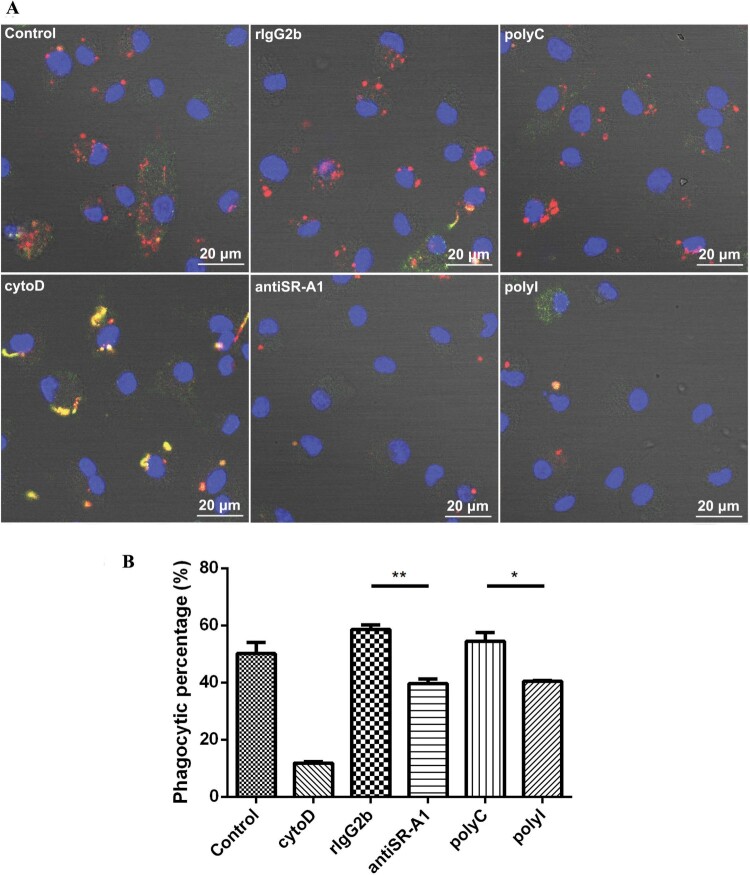
PolyI and SR-A1 monoclonal antibody (anti-SR-A1) exhibited the inhibition to the phagocytosis of *L. interrogans* strain 56606v by mouse BMDMs. BMDMs were incubated with active *L. interrogans* strain 56606v in FBS-free medium absence or presence of cytoD (20μM), polyI (100μg/mL) and anti-SR-A1 (30μg/mL). Corresponding concentrations of polyC and rat IgG2b (rIgG2b) isotypes were added as controls. Rabbit anti-*L. interrogans* strain 56606v was treated as a specific primary antibody, while FITC-conjugated or TRITC-conjugated anti-rabbit IgG as a secondary antibody were used before and after permeabilization. Confocal microscopic images showed leptospires inside (red) or outside (yellow) of BMDMs **(A)**. Phagocytic percentages of BMDMs phagocytizing *L. interrogans* were calculated and statistically analysed by variance **(B)**. These data were expressed as the mean ± SEM from at least three experiments. *P < 0.05, **P < 0.01.

To determine the contribution of SR-A1 to the nonopsonic-phagocytosis of *L. interrogans* by murine BMDM, we investigated the inhibition of leptospires uptake by SR-A1 ligand polyI (a general SR inhibitor) and anti-SR-A1 2F8 (an anti-SR-A1 monoclonal antibody) without opsonins. Indirect immunofluorescence staining was used to detect the leptospires inside (red) and outside (merged in yellow for both binding of FITC-conjugated and TRITC-conjugated antibodies before and after permeabilization) of macrophages. Confocal microscopic images showed that polyI and anti-SR-A1 could significantly inhibit the phagocytosis of active *L. interrogans* by BMDM cells compared to the corresponding controls polyC and rIgG2b (Figure 2). Similar results were found in inhibition experiments of phagocytosis of inactive *L. interrogans* by BMDM cells (Supplementary Figure 1). The FCM method was employed further to confirm our findings (Supplementary Figure 1). PM cells from another murine strain, C57BL/6 mice, also verified the inhibitory effect of SR-A1 chemical inhibitors on phagocytosis to *Leptospira* (Supplementary Figure 2). The results showed that inhibitors of SR-A1 could significantly reduce phagocytosis rates of *Leptospira* by macrophages. However, inhibition of the mannan receptor which was not significantly regulated by leptospiral stimulation, exhibited no inhibition to phagocytosis in *L. interrogans* strain 56606v, indicating this receptor did not participate in this process (Supplementary Figure 3).

**Figure 3.**
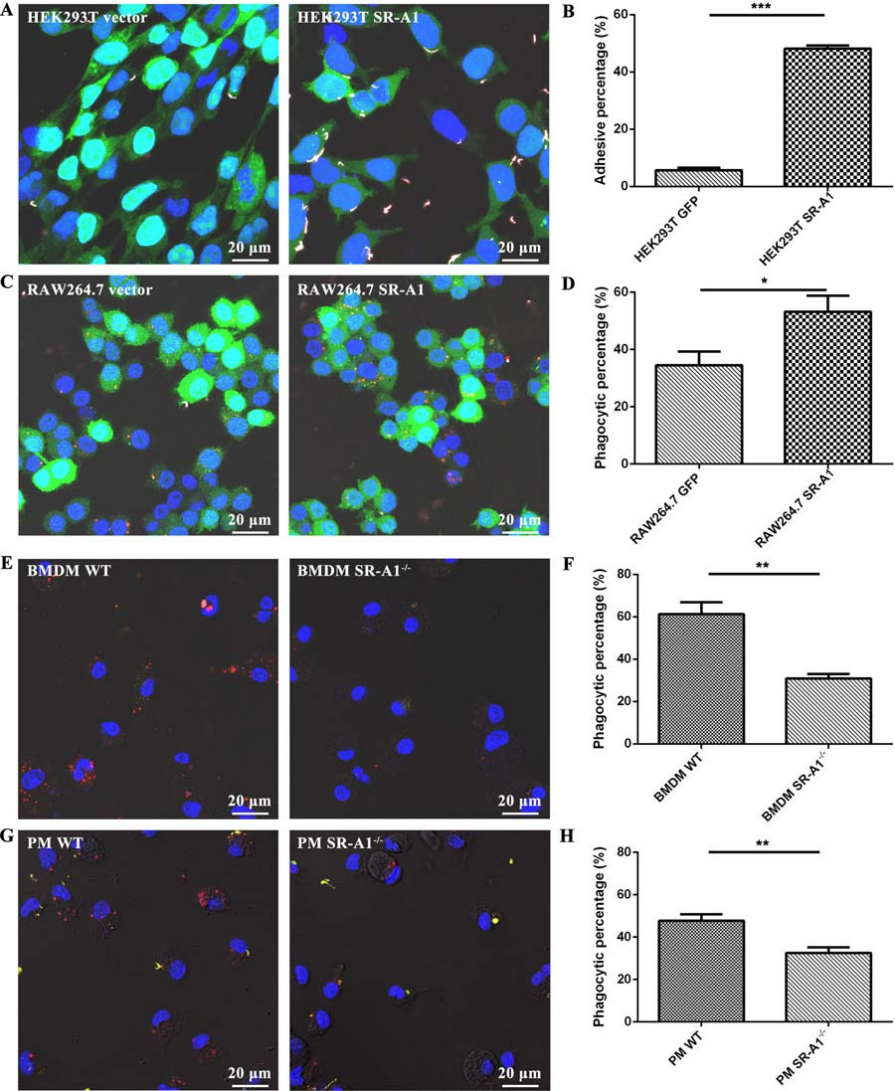
SR-A1 enhanced *L. interrogans* adhesion and phagocytosis. Lentivirus transfected HEK293 T vector cells and HEK293 T SR-A1 cells **(A)**, RAW264.7 vector cells and RAW264.7 SR-A1 cells **(C)** with GFP expression, WT and SR-A1^-/-^ BMDMs **(E)** or PMs **(G)** were incubated with *L. interrogans* strain 56606v at MOI = 50 for 1 h in medium without FBS, respectively. Rabbit anti-*L. interrogans* strain 56606v was used as a specific primary antibody, while Alexa Fluor 647-conjugated or TRITC-conjugated anti-rabbit IgG as a secondary antibody were used before and after permeabilization, respectively. Confocal microscopic images showed leptospires inside (red) or outside (white/yellow) of cells. Positive rates of *Leptospira* adhesive cells or phagocytic cells were calculated, respectively **(B, D, F, H)**. These data were expressed as the mean ± SEM from at least three experiments. *P < 0.05, **P < 0.01, ***P < 0.001.

To further verify the role of SR-A1 in *L. interrogans* adhesion and phagocytosis, HEK293 T or RAW264.7 cells transfected with SR-A1 vector were constructed. Cell surface overexpression of SR-A1 was verified by the FCM method (Supplementary Figure 4). SR-A1-transfected HEK293 T cells showed significantly increased adhesion of inactive *L. interrogans* compared to mock cells, with a large amount of fluorescent merged leptospires visible attaching to the cell membrane (Figure 3A). Overexpression of SR-A1 in RAW264.7 cells also showed higher phagocytic rates than mock cells (Figure 3B). These results confirmed the SR-A1 function in adhesion and phagocytosis of *Leptospira*.

SR-A1 gene knockout mice were used to characterize further the role of SR-A1 in the phagocytosis of leptospires. SR-A1^-/-^ BMDMs phagocytosed about 50% less *L. interrogans* than WT BMDMs did (Figure 3E and F). Consistent results were also achieved in PMs from SR-A1^-/-^ and WT mice (Figure 3G and H). These data indicated that SR-A1 is an important murine macrophage receptor for nonopsonic-phagocytosis of *L. interrogans*.

### SR-A1^-/-^ macrophages were deficient in phagocytosis of *L. interrogans in vivo*

**Figure 4.**
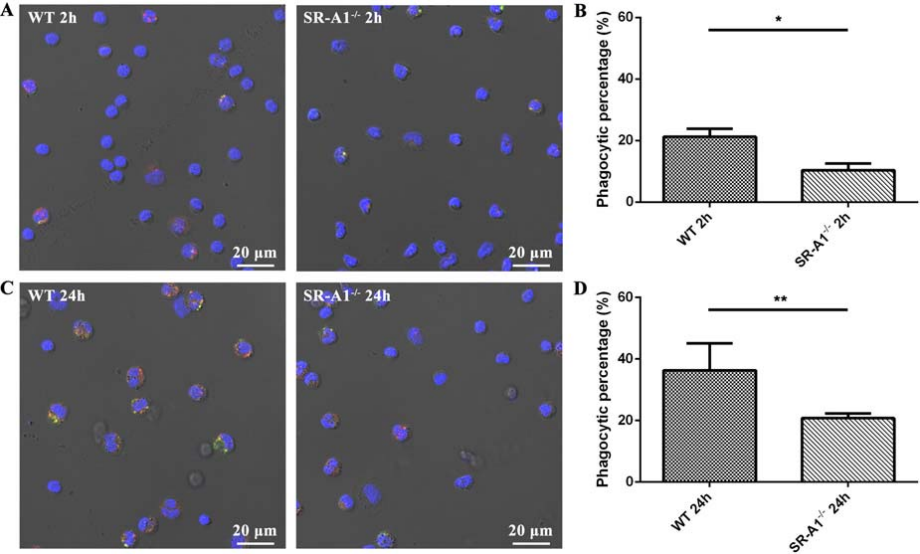
SR-A1^-/-^ PMs were deficient in phagocytosis of *L. interrogans* strain 56606v *in vivo*. WT or SR-A1^-/-^ mice were infected by IP injection of 2 × 10^8^ bacteria per mouse. PMs were harvested at 2 and 24 hpi, respectively. Rabbit anti-*L. interrogans* strain 56606v was used as a specific primary antibody, while FITC-conjugated or TRITC-conjugated anti-rabbit IgG as a secondary antibody were used before and after permeabilization, respectively. Confocal microscopic images showed leptospires inside (red) or outside (yellow) of PMs at 2 hpi **(A)** and 24 hpi **(C)**. Phagocytic percentages were calculated and statistically analysed **(B, D)**. These data were expressed as the mean ± SEM from at least three experiments. **P < 0.01.

To evaluate the phagocytosis ability of SR-A1 in the presence of opsonin *in vivo*, we inoculated leptospires in the peritoneal cavity of WT and SR-A1^-/-^ mice. PMs were harvested at 2 and 24 hpi, and leptospires were stained and detected by confocal microscopy. The result showed that WT PMs have higher phagocytic rates than SR-A1^-/-^ PMs (Figure 4). Our results showed that SR-A1 also plays a significant role in the phagocytosis of *L. interrogans in vivo*.

### SR-A1^-/-^ mice were more resistant to *L. interrogans* infection compared with WT mice

**Figure 5.**
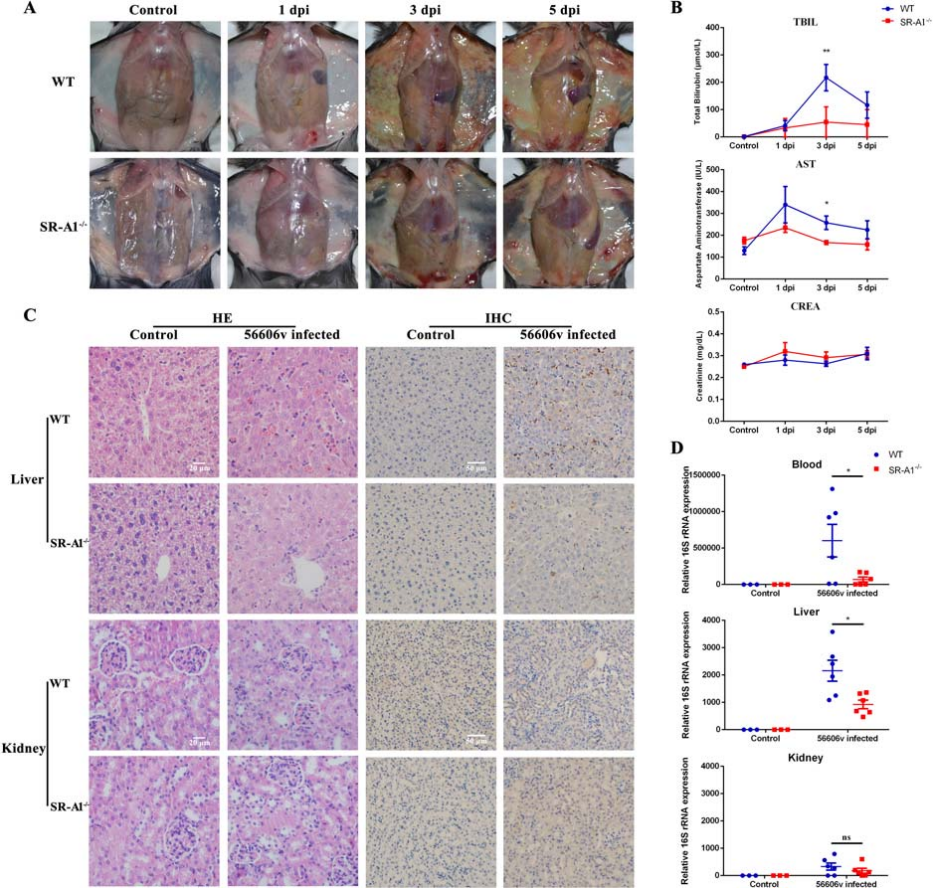
SR-A1^-/-^ mice were more resistant to *L. interrogans* infection as compared to WT mice*.* WT or SR-A1^-/-^ mice (*n *= 3 per time point group, except for *n *= 6 at 1 dpi in leptospiral loads detection) were infected by IP injection of 2 × 10^8^ bacteria per mouse. **(A)** Gross observation of jaundice and hemorrhage in abdominal and subcutaneous tissue was illustrated. **(B)** The levels of serum total bilirubin (TBIL), aspartate transaminase (AST) and creatinine (CREA) on behalf of liver and kidney function were measured by UniCel DxC 800 Synchron autoanalyzer. **(C)** H&E staining in different organs was performed for histopathological analysis, and the leptospires in organs were detected by immunohistochemistry. **(D)** The leptospiral loads in the blood, liver and kidney of mice at 1 dpi were determined through qPCR method of bacterial 16S rRNA. The results were calculated by mean ± SEM from three or six mice per time point and were representative of three independent experiments. *P < 0.05, **P < 0.01, NS no significance between WT and SR-A1^-/-^ mice.

It has been reported that SR-A1^-/-^ mice were more susceptible to some bacterial infections than WT mice [16,17,27]. This is most likely due to SR-A1 mediating opsonin-independent phagocytosis of bacteria, which plays a critical role in host defense against bacterial infections. To determine whether SR-A1 also plays an important role in defense against *L. interrogans in vivo*, WT and SR-A1^-/-^ mice were infected by intraperitoneal injection of 2 × 10^8^ *L. interrogans* strain 56606v, and the pathological lesions were examined at 1, 3 and 5 days post-infection. None of the WT or SR-A1^-/-^ mice died from the infection during the study. Strikingly, infected WT mice developed more obvious jaundice and subcutaneous hemorrhage than SR-A1^-/-^ mice (Figure 5A). Two liver serological markers, total bilirubin (TBIL) and aspartate transaminase (AST), were found elevated in infected mice with significantly higher in WT than in SR-A1^-/-^ mice. In contrast, the kidney serological marker creatinine (CREA) remained at normal levels in both WT and SR-A1^-/-^ mice (Figure 5B).

H&E staining revealed reduced lesions of acute leptospirosis in the liver of SR-A1^-/-^ mice compared with that of WT mice (Figure 5C). The loss in the liver architecture of WT mice was observed along with hepatocyte focal necrosis, hemorrhage and Kupffer cell hyperplasia. But only a mild focal hemorrhage was observed in SR-A1^-/-^ mice. There were no severe lesions in the kidney tissues of both mice (Figure 5C). None of the pathological changes was observed in PBS-inoculated control animals.

The leptospiral loads were determined by estimates of the copy number of *Leptospira* 16S rRNA in blood and tissues. A significantly lower bacterial burden was observed at 1 dpi in the blood and liver of the infected SR-A1^-/-^ mice than in WT mice (Figure 5D). Leptospires were also detected in different organs by immunohistochemistry (Figure 5C). Consistent with the 16S rRNA detection, SR-A1^-/-^ mice showed lower bacterial burdens in the liver than in those of WT mice (Figure 5C). There was no difference in leptospiral loads in the kidney tissues between infected SR-A1^-/-^ and WT mice.

**Figure 6.**
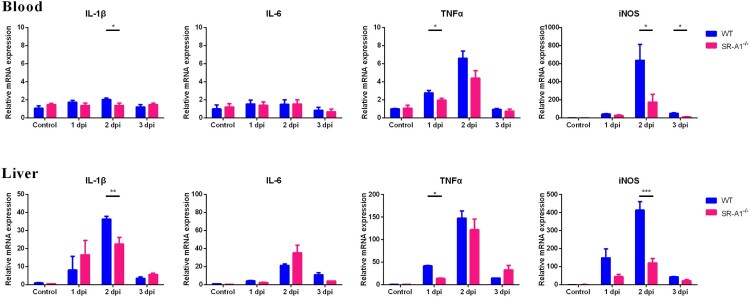
SR-A1^-/-^ mice illustrated reduced IL-1β, TNFα and iNOS response to *L. interrogans* infection as compared to WT mice*.* WT or SR-A1^-/-^ mice (*n *= 3 per time point group) were infected by IP injection of 2 × 10^8^ *L. interrogans* strain 56606v per mouse. Cytokines expression in the blood and liver of mice at 1-3 dpi were determined through qPCR method. The results were calculated by mean ± SEM from three mice per time point, and were representative of three independent experiments. *P < 0.05 between WT and SR-A1^-/-^ mice.

In the detection of cytokines, we found that the expression of iNOS and pro-inflammatory factors such as IL-1β and TNFα was significantly reduced in the blood and liver of SR-A1^-/-^ mice (Figure 6). IL-6 expression in vivo was not affected by SR-A1. There was no severe inflammation in kidney tissues from either group (data not shown) and were consistent with pathology experiments in Figure 5.

### SR-A1 increase live *L. interrogans* loads in macrophages *in vitro*

Decreased *L. interrogans* burden in SR-A1^-/-^ mice might seem counterintuitive since SR-A1 increases *Leptospira's* uptake by macrophages. SR-A1^-/-^ mice cleared *Leptospira* from the liver and blood more efficiently compared with WT mice, suggesting that SR-A1 contributes to *Leptospira* infection pathophysiology. Previous studies reported that *Leptospira* could survive and replicate in macrophages and even be transported to organs by infected macrophages [28]. In this study, immunohistochemical results showed that macrophage recruitment of *Leptospira* infected WT mice in liver tissue was significantly higher than that of SR-A1^-/-^ mice (Supplementary Figure 6), which was consistent with the previous findings that SR-A1 might influence macrophage recruitment [29]. Thus, we postulate that SR-A1 might enhance the live leptospires load in macrophages and contribute to leptospiral dissemination in organs.

To test this hypothesis, we performed the *in vitro* experiment to detect leptospiral survival within macrophages. We found a small proportion of ingested leptospires survived in macrophages at 72 hpi (Supplementary Figure 5), although most internalized *Leptospira* were killed at 48 hpi. The live leptospires load was higher in WT macrophages than in SR-A1^-/-^ macrophages at 72 hpi (Supplementary Figure 5). These results indicated that SR-A1 increases the live leptospires load in macrophages and might promote the dissemination of *Leptospira* in organs.

### SR-A1 is essential for intracellular receptor-mediated cytokine and inflammation mediator secretion in macrophages infected by *Leptospira in vitro*

**Figure 7.**
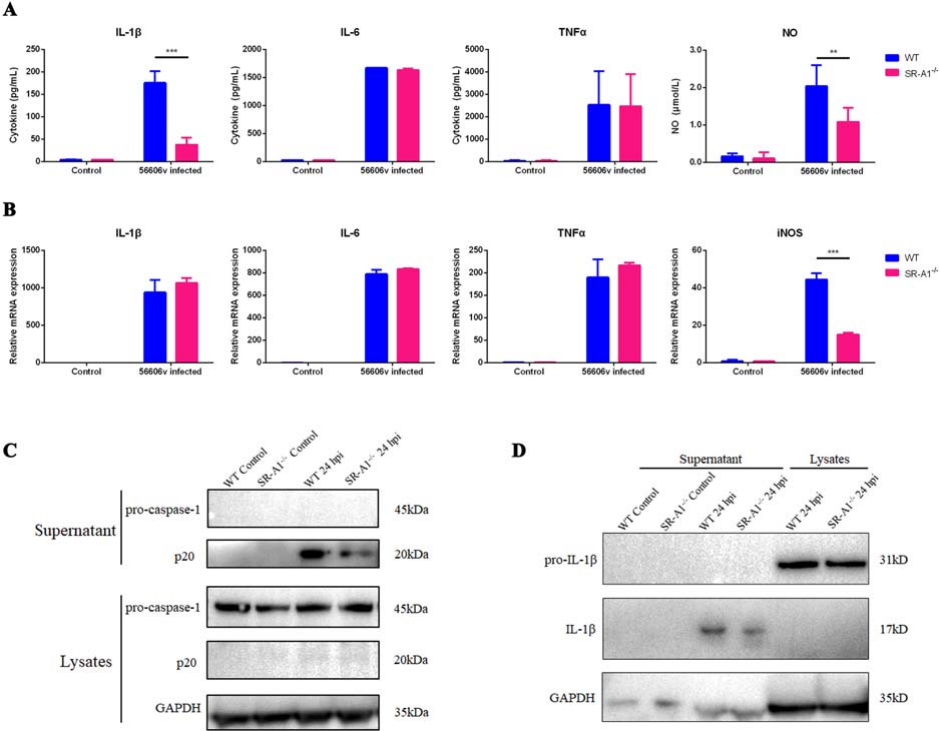
SR-A1 enhance the intracellular receptor activation in *L. interrogans* infected macrophages *in vitro*. PMs from WT or SR-A1^-/-^ mice were infected by *L. interrogans* strain 56606v as MOI* *= 100 *in vitro*. **(A)** The cytokine level of the supernatant at 1 dpi were quantified by ELISA kits from R&D Systems, and contemporaneous NO levels was tested immediately via the Griess reaction. **(B)** Cytokines and iNOS expression of PMs at 3 hpi were quantified through the RTqPCR method. Caspase-1 **(C)** and IL-1β **(D)** extracted from supernatants and lysates at 1 dpi were quantified by Western blot. Precursor and spliceosome were distinguished by molecular weight. These data were expressed as the mean ± SEM from at least three experiments. **P < 0.01, ***P < 0.001.

*In vivo* studies showed that WT mice had higher *Leptospira* loads and increased inflammatory responses than those of SR-A1^-/-^ mice. Exacerbated inflammatory responses in WT mice might be due to the consequence of high *Leptospira* loads or the inflammatory process triggered by SR-A1. Prior studies suggested that pathogen internalization via SR-A1 prevents sustained sensing and response by surface TLRs, increasing the intracellular immune responses through intracellular receptors, such as NOD1, NALP3 and TLR3 [30]. To evaluate whether SR-A1 has a direct role in modulating inflammatory responses, we measured NO and cytokines expression using WT and SR-A1^-/-^ PMs infected with *Leptospira in vitro.* We performed ELISA test of supernatants from PMs stimulated with *L. interrogans* as MOI 100 at 1 dpi *in vitro*. NO and IL-1β expression levels were significantly decreased in SR-A1^-/-^ cells (Figure 7A), consistent with cytokine expression *in vivo* (Figure 6). However, IL-6 and TNFα expression *in vitro* were not affected by SR-A1 (Figure 7A). The transcriptional level of TNFα, IL-6, IL-1β and iNOS mRNA in *Leptospira* infected macrophages were also detected. The results showed that SR-A1^-/-^ macrophages produced significantly less iNOS mRNA compared with WT macrophages (Figure 7B). However, the mRNA levels of IL-1β, IL-6 and TNFα were not affected by SR-A1 (Figure 7B).

To understand the difference in protein and mRNA level of IL-1β expression between infected WT and SR-A1^-/-^ macrophages, we assessed the inflammasome activation. It was found that spliceosome of caspase-1 (P20) in the supernatant was upregulated by *Leptospira* stimulation, and P20 of SR-A1^-/-^ PMs was significantly decreased compared with that of WT PMs (Figure 7C). Consistently, pro-IL-1β (IL-1β) spliceosome in the supernatant was significantly reduced in SR-A1^-/-^ PMs (Figure 7D). These results indicated that lower protein level of IL-1β in infected SR-A1^-/-^ PMs was related to the reduced inflammasome caspase-1 activation.

## Discussion

Scavenger receptor A1, known as the macrophage scavenger receptor that binds and traffics a variety of microbial ligands, plays multiple roles in inflammation, innate immunity and host defense [19]. SR-A1 is a phagocytic receptor for a variety of gram-positive and gram-negative bacteria [15,16,18]. However, some bacteria, such as *Escherichia coli* K12, *Pseudomonas aeruginosa* PA14, *Streptococcus agalactiae* and *Streptococcus pyogenes*, were described as being uptake by macrophages through an SR-A1-independent way [31,32]. The surface M protein and the sialylated polysaccharide capsule of streptococci prevent the SR-A1 mediated phagocytosis by macrophages [33]. Unlike other typical gram-negative bacteria, *Leptospira* is a bacterium with unique evolutionary and structural characteristics. Our understanding of the receptors involved in the process of *Leptospira’s* phagocytosis is still minimal. Several host receptors such as CR3 receptor and β2 integrin have been proved to be involved in the phagocytosis of *L. interrogans* [10,11], while some PRRs, like the mannose receptors (MRs) as shown above in our study, are less likely to participate in this process.

This study demonstrates that SR-A1 is a primary nonopsonic phagocytic receptor for *Leptospira* on murine macrophages. We utilized SR-A1 inhibitor, SR-A1 blocking antibody, SR-A1^-/-^ murine macrophages, and SR-A1 overexpressed cells to examine the interaction of SR-A1 and *Leptospira*. The application of SR-A1 inhibitor polyI, lacks specificity in binding SR-A1, while the SR-A1^-/-^ macrophages induce compensatory up-regulation of alternative receptors, causing misinterpretation of the results. To avoid these issues, we used the SR-A1 specific antibody to block the SR-A1 binding function. Our results showed that the *Leptospira* phagocytosis ratio by SR-A1^-/-^ macrophages are down-regulated to appropriately 50%. A similar result was also obtained when WT macrophages were treated with SR-A1 blocking antibody. These results indicate that SR-A1 plays a vital role in phagocytosis *in vitro*.

SR-A1^-/-^ mice were used to investigate SR-A1 phagocytosis function *in vivo*. After intraperitoneal infection, invading *Leptospira* can be opsonized by natural antibodies and complement in the plasma, promoting their phagocytosis through CR3 receptors. Our results showed that WT PMs have higher phagocytic rates than that of SR-A1^-/-^ PMs (Figure 4). The phagocytic function of SR-A1 was not concealed by the effect of opsonic receptors in the peritoneal fluid of experimentally infected animals, suggesting that SR-A1 also plays a significant role in the phagocytosis of *Leptospira in vivo*.

Earlier works have suggested that the contribution of SR-A1 to host defense of bacterial infection varied with the specific strain [27,34]. It was shown previously that SR-A1^-/-^ mice are more susceptible to *Listeria monocytogenes, Neisseria meningitidis* and *Streptococcus pneumonia* infection than WT [16,17,27]. In contrast, other studies indicated that SR-A1^-/-^ mice have a decreased susceptibility to infection with *Mycobacterium tuberculosis, Pneumocystis carinii* and polymicrobial sepsis [34–36]. Although studies have clearly demonstrated that SR-A1 is important in *Leptospira* uptaken by macrophages in the peritoneal cavity, it remains to be tested *in vivo* if the high phagocytosis plays a vital role in host defense. Our acute leptospirosis murine model provides a powerful tool to clarify the question [21]. Using this model, mice intraperitoneally injected with leptospires displayed high bacterial loads in blood and tissues and apparent clinical symptoms of leptospirosis. Macrophages have been indicated to be the primary infiltrating and anti-*Leptospira* phagocytes during *Leptospira* infection [37]. Enhancing the phagocytic activity of macrophages seems to increase the ability to fight infection. Surprisingly, leptospiral infected WT mice presented more apparent jaundice, subcutaneous hemorrhage, and higher bacteria burden in blood and tissues than that of SR-A1^-/-^ mice, suggesting that *Leptospira* can exploit SR-A1 to promote their dissemination and cause apparent symptoms in the host.

Multiple pathogens have evolved strategies to regulate macrophages activation and responses. *Mycobacterium marinum* and *Mycobacterium leprae* were reported taking advantage of macrophages as vehicles for bacterial dissemination [38,39]. Clay *et al*. showed that while depleting macrophages led to higher *Mycobacterium marinum* burdens and increased host death, it also decreased the dissemination of pathogens into deeper tissue [38]. This was referred to as a “dichotomous role” of macrophages. In case of *L. interrogans*, previous studies using the zebrafish embryos model confirmed that infected macrophages participate in *L. interrogans* dissemination [40]. Although *L. interrogans* is usually considered to be extracellular pathogens, they can survive and even replicate in murine macrophages [28,41]. Macrophages were capable of killing opsonized leptospires effectively but had little bactericidal activity against nonopsonized *L. interrogans*, and intact leptospires could be found in the cytosol [42,43]. In this study, the survival of *L. interrogans* in peritoneal macrophage showed that a small proportion of ingested *L. interrogans* survived in macrophages 72 hpi, and live *L. interrogans* loads were higher in WT macrophages than in SR-A1^-/-^ macrophages (Supplementary Figure 5). This may be due to the contribution of SR-A1-related nonopsonic phagocytosis. Our findings suggest that infected macrophages might participate in leptospiral dissemination, as a higher *Leptospira* load in macrophage following a higher *Leptospira* burden in organs. Another characteristic of SR-A1, cell adhesion and surface localization, which are essential for macrophage recruitment to the sites of tissue infected [29], might be partly responsible for the higher bacteria load in the tissue of WT mice. In this study, WT mice had more macrophage recruitment in liver tissue than that of SR-A1^-/-^ mice (Supplementary Figure 6). It further suggested that infected macrophages might exploit SR-A1 to promote leptospires spread to target organs. However, our proposed mechanism was different from previous studies reporting that phagocytes depleted by clodronate liposomes led to enhanced leptospiral burdens in the acute phase [44]. One likely explanation for this difference might be that infected macrophages are not the sole mechanism for dissemination. Elucidation of the comprehensive mechanisms for *Leptospira* dissemination will need further study.

In this study, SR-A1^-/-^ mice presented more apparent symptoms and tissue damage than WT mice. Our previous study suggested that apparent hemorrhage is more related to the overexpression of iNOS and proinflammatory cytokines than at the higher burden of *Leptospira* [21]. To analyse the possible correlation between the SR-A1 and inflammatory response, we evaluated the TNFα, IL-6, IL-1β and iNOS level in WT and SR-A1^-/-^ mice. We observed markedly higher levels of TNFα, IL-1β and iNOS mRNA in the liver and blood of WT mice compared to SR-A1^-/-^ mice in early stages and correlation with the degree of clinical symptoms (Figure 6). *In vitro* study using peritoneal macrophages stimulated with *Leptospira* showed similar results with higher cytokines produced by WT cell compare with that of SR-A1^-/-^ macrophages (Figure 7). It seems that an increased inflammatory response was not only because of the higher *Leptospira* burden in the host but also due to SR-A1 activity.

In addition to its scavenging function, SR-A1 modulates inflammatory responses. However, the mechanism of how SR-A1 regulates inflammation responses is still unclear. Some contradictory results were found by several studies suggesting both pro-inflammatory and anti-inflammatory roles of SR-A1 [30,35,45–47]. One theory proposed by Subhankar *et al*. is that SR-A1 attenuates TLR4-driven proinflammatory cytokine responses by endocytic scavenging of LPS from the extracellular environment, whereas SR-A1 enhances intracellular immune responses through intracellular receptors, such as NOD1, NALP3 and TLR3 [30]. Pathogen ligands could activate macrophages by binding to TLR4 and triggered two major pro-inflammatory signalling pathways, including MyD88-dependent pathways triggered at the plasma membrane and TRIF-dependent pathways in endosomes [48]. It was recently shown that leptospiral LPS signalling through TLR2 escapes the TLR4/TRIF/NO pathway [48]. However, in the case of *L. interrogans* strain 56606v, our previous work showed this strain possesses an LPS atypically only signalling through TLR4 [21], which may trigger the TRIF/NO pathway. The induction of TNFα and IL-6 was mainly MyD88-dependent, and the results indicated that surface receptor-mediated cytokines induced by *Leptospira* are unaffected by SR-A1. On the other hand, the production of NO, primarily dependent on the intracellular receptors, such as the TRIF adaptor or TLR3, was upregulated by SR-A1. In addition, the ELISA results also showed SR-A1 upregulated IL-1β secretion. IL-1β secretion requires TLR-mediated induction of pro-IL-1β, followed by activation of the NALP3 inflammasome and cleavage of pro-IL-1β to mature IL-1β [49]. IL-1β mRNA levels, representative of TLR-mediated induction, were not affected by the presence of SR-A1, indicating the increased IL-1β cytokine secretion was mainly due to the activation of NALP3/caspase-1-mediated cleavage of pro-IL-1β to mature IL-1β [46]. This study found that spliceosome of caspase-1 and IL-1β in supernatants of *Leptospira* infected SR-A1^-/-^ PMs was significantly reduced than that of WT PMs (Figure 7). Our results suggested that *Leptospira* internalization via SR-A1 is involved in intracellular receptor-mediated inflammatory cytokine and mediator secretion.

**Figure 8.**
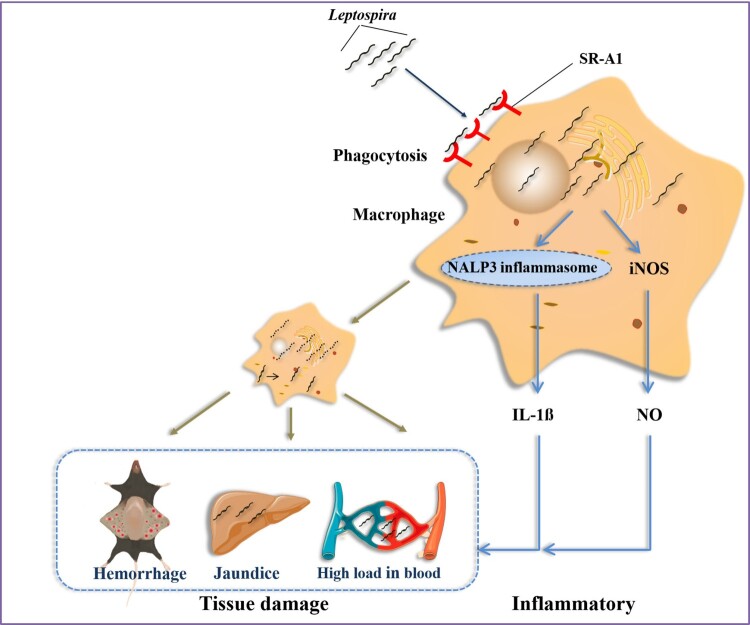
Strategies employed by *Leptospira* to take advantage of SR-A1 contributing to infection. In *Leptospira*-infected mice, macrophages play a controversial role in controlling leptospires in the initial stage of infection. SR-A1 on macrophages binds *Leptospira*, mediating the phagocytosis of *Leptospira*. The majority of internalized *Leptospira* were killed, whereas a small proportion of ingested *Leptospira* survived in macrophages. SR-A1 mediated phagocytosis increased *Leptospira* load in macrophages, which may trigger the intracellular receptor excessive activation and enhance the secretion of IL-1β and NO, and consequently cause apparent jaundice, subcutaneous hemorrhage and high *Leptospira* burden in blood and tissues.

Pathogens have evolved numerous complex mechanisms to exploit loopholes in the immune system to gain advantages over host immune defenses. Understanding the processes that pathogens manipulate immune responses are critical for controlling the infection. Our study reveals that although the host is beneficial in the uptake of *Leptospira* by macrophages, SR-A1 might be exploited by *Leptospira* to modulate inflammatory activation and increase the susceptibility of infection in the host. Leptospiral strategies to take advantage of SR-A1 contributing to infection are depicted in Figure 8. This study suggests the potential of utilizing SR-A1 inhibitor in the treatment of leptospires infection.

## Supplementary Material

Supplemental_Materials_-_revise2_-_clean.docx
