# Supplementary material for: Scavenger receptor A1 participates in uptake of Leptospira interrogans serovar Autumnalis strain 56606v and inflammation in mouse macrophages
Source: Emerg Microbes Infect. 2021 May 18;10(1):939–53. doi: 10.1080/22221751.2021.1925160 (PMC8153709; doi:10.1080/22221751.2021.1925160)
Supplement: Supplemental_Materials_-_revise2_-_clean.docx [file TEMI_A_1925160_SM9294.docx]

**Supplemental Materials**

**Materials and Methods**

**FITC labeling of bacterial strains**

Phosphate buffered saline (PBS) washed bacteria were diluted in 1 mL of carbonate buffer solution (CBS) containing 10 μM FITC (Invitrogen, USA). The mixture for labeling was incubated for 30 min at room temperature, protected from light. When using inactivated bacteria, the bacterial suspension was then fixed for 1 h at 4°C with 4% paraformaldehyde. *L. interrogans* were washed and counted in a Petroff-Hauser chamber under dark-field microscopy and then were suspended in RPMI 1640 at a particular concentration for the experiment.

**Adhesion and phagocytosis of leptospires by flow cytometry (FCM) analysis**

Fixed leptospires were pre-stained by FITC, as mentioned above. Each flow tube of 1×10^6^ macrophages was incubated with bacteria as MOI=10 and rotated at 10 rpm in cell culture condition of 37°C. After infection, trypan blue was used to quench the fluorescence of *L. interrogans* outside the cell membrane. FCM of bacteria *in vivo* and cytoD treated cells were used to confirm quenching. Cells were harvested and fixed with 1% paraformaldehyde for FCM (Caliber, Becton Dickinson, USA) detection. A total of 10,000 cells were counted, and the percentages of adhesion and phagocytosis were calculated as the number of positive cells with respect to the total events counted.

**Western blot analysis of SR-A1 overexpression**

Total protein extracted from transfected HEK293T and RAW264.7 cells was resolved by SDS-PAGE and electrotransferred to polyvinylidene fluoride (PVDF) membranes (Millipore, USA), and was blocked with 5% nonfat milk (Sangon Biotech, China). SR-A1 primary antibody (rabbit monoclonal antibody, Abcam, USA) and HRP-conjugated secondary antibody (anti-rabbit IgG, Beyotime, China) were used to detect the SR-A1 expression. The blots were visualized with a chemiluminescent substrate (Thermo Scientific, USA) and exposed to ImageQuant LAS4000 (GE Healthcare, USA).

**FCM detection of cell surface overexpression of SR-A1**

Cell surface SR-A1 expression in transfected HEK293T and RAW264.7 cells were detected through the FCM method. Cells were harvested and fixed with 1% paraformaldehyde without permeabilization. SR-A1 primary antibody (rabbit monoclonal antibody, Abcam, USA) and Alexa Flour 647-conjugated secondary antibody (anti-rabbit IgG, Abcam, USA) were used to detect the SR-A1 expression on the cell surface.

**Detection of survival of *L. interrogans* strain 56606v in leptospires infected WT and SR-A1^-/-^ peritoneal macrophages (PMs)**

WT and SR-A1^-/-^ PMs were seeded by 1×10^6^ cells per well in a 12-well plate, and then were infected by *L. interrogans* strain 56606v as MOI=100 for 2 h *in vitro*. 100 μg/mL gentamicin was used for 1 h to kill the extracellular leptospires. Leptospiral RNA from cells of 1 hpi to 72 hpi was extracted and quantified by real-time PCR, as mentioned in the main text.

**Macrophages recruitment in the liver after *L. interrogans* stimulation *in vivo***

WT and SR-A1^-/-^ mice were infected with 2×10^8^ leptospires via the intraperitoneal route. Livers from 1 to 2 dpi of infected mice were fixed in neutral buffered 4% formaldehyde, followed by embedment of the tissues in paraffin. Sections were immunohistochemically stained for macrophage-specific F4/80 rabbit antibody of 1:600 dilution (Servicebio, China). Macrophages were counted in at least 10 HP fields, and the average number of F4/80 expressing cells per HP field was calculated.

**Results**

**Tables**

**Supplementary Table 1.** Primers used to detect receptors and cytokines expression.

| **Primer target** | **Sequence 5' to 3'** | **Acession** | **Position** |
| --- | --- | --- | --- |
| *SR-A1* | F: ATGAACAAGAGGATGCTGAC | NM_031195.2 | 99-118 |
|  | R: CAAACACAAGGAGGTAGAGAG |  | c257-c237 |
| *CR3* | F: ATGGACGCTGATGGCAATACC | NM_001082960.1 | 1499-1519 |
|  | R: TCCCCATTCACGTCTCCCA |  | c1704-c1686 |
| *Fcgr1* | F: AGGTTCCTCAATGCCAAGTGA | NM_010186.5 | 298-318 |
|  | R: GCGACCTCCGAATCTGAAGA |  | c495-c476 |
| *MR* | F: CTCTGTTCAGCTATTGGACGC | NM_008625.2 | 144-164 |
|  | R: CGGAATTTCTGGGATTCAGCTTC |  | c275-c253 |
| *Clec* | F: GGAAAGTCATTCCAGACCCA | NM_010819.4 | 799-818 |
|  | R: AAGACGCCATTTAACCCACA |  | c719-c700 |
| *Stab1* | F: CCACTCCAAATGAAGACTTG | NM_138672.2 | 5790-5809 |
|  | R: CTACTCATGTGGTTACGATTC |  | c5980-c5960 |
| *Stab2* | F: GCTGCAAGTCCTCATGTCCT | NM_138673.3 | 7024-7043 |
|  | R: TTCTGTGGCACAAACAGGGT |  | c7167-c7148 |
| *Marco* | F: TTAGCAGCTATGGAGGTGGC | NM_010766.3 | 1656-1675 |
|  | R: GACACACTGATGACCTCTCGG |  | c1834-c1814 |
| *Siglec1* | F: GTCTCCAGGAAGGTGGTCAG | NM_011426.3 | 1919-1938 |
|  | R: CAGGGCTGATACTGGCTTCT |  | c1832-c1813 |
| *TNFα* | F: CCTGTAGCCCACGTCGTAG | NM_013693.3 | 438-456 |
|  | R: GGGAGTAGACAAGGTACAACCC |  | c585-c564 |
| *IL1β* | F: GAAATGCCACCTTTTGACAGTG | NM_008361.3 | 96-117 |
|  | R: TGGATGCTCTCATCAGGACAG |  | c211-c191 |
| *IL6* | F: TAGTCCTTCCTACCCCAATTTCC | NM_031168.1 | 519-541 |
|  | R: TTGGTCCTTAGCCACTCCTTC |  | c594-c574 |
| *iNOS* | F: ACATCGACCCGTCCACAGTAT | NM_001313921.1 | 220-240 |
|  | R: CAGAGGGGTAGGCTTGTCTC |  | c396-c377 |
| *GAPDH* | F: AGGTCGGTGTGAACGGATTTG | NM_008084.3 | 248-268 |
|  | R: TGTAGACCATGTAGTTGAGGTCA |  | c370-c348 |

^a^ F, forward primer; R, reverse primer

**Figures**


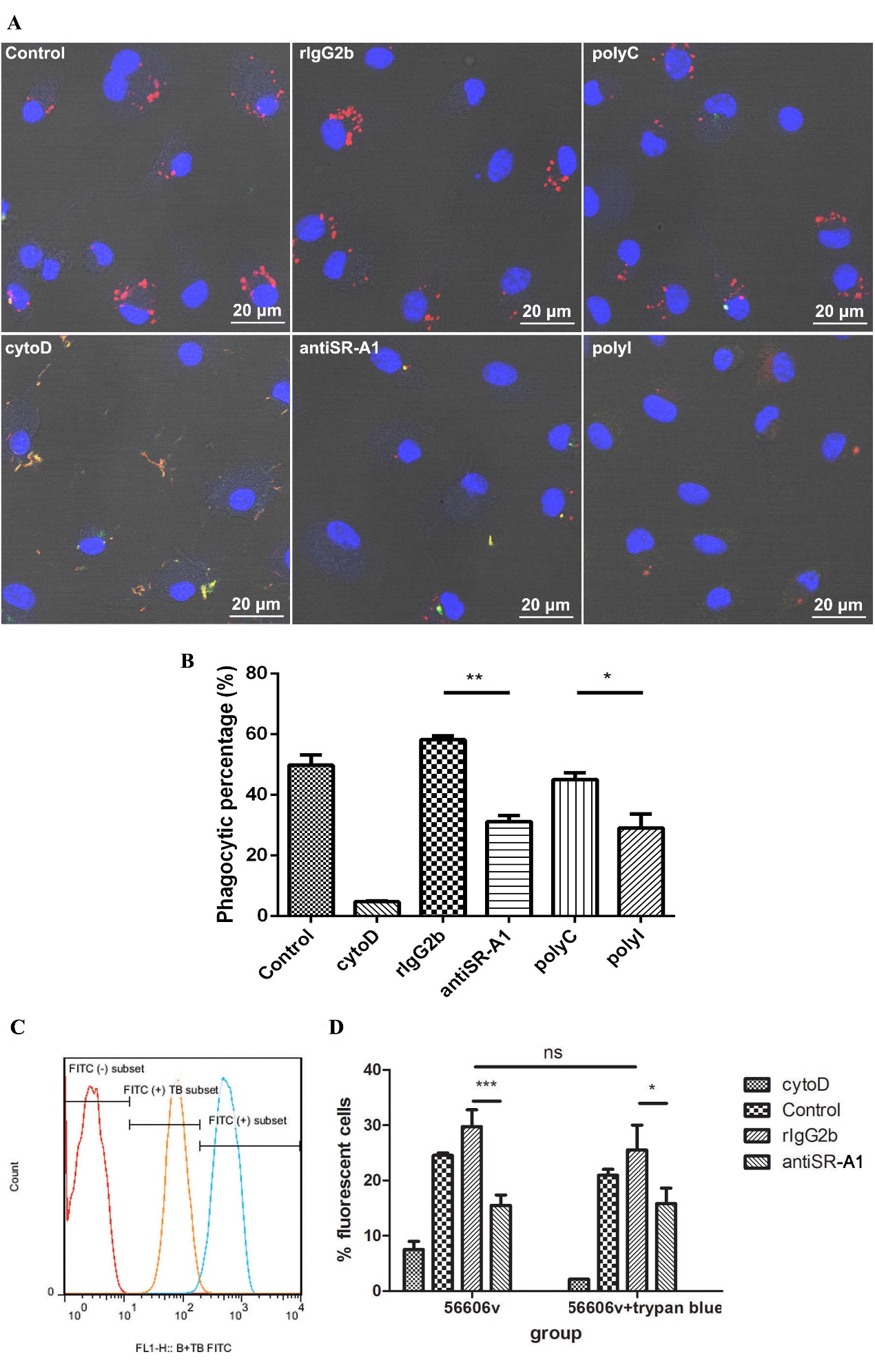


**Supplementary Fig. 1.** PolyI and SR-A1 monoclonal antibody (anti-SR-A1) exhibited the inhibition to phagocytosis of alive *L. interrogans* strain 56606v by mouse bone marrow-derived macrophages (BMDMs).

BMDMs were incubated with inactive *L. interrogans* strain 56606v at MOI=10 in FBS-free medium absence or presence of cytoD (20μM), polyI (100μg/mL) and anti-SR-A1 (30μg/mL). Corresponding concentrations of polyC and rat IgG2b (rIgG2b) isotypes were added as controls. **(A)** Rabbit anti-*L. interrogans* strain 56606v was treated as specific primary antibody, while FITC-conjugated or TRITC-conjugated anti-rabbit IgG as secondary antibody were used before and after permeabilization, respectively. After observations by confocal microscopy, **(B)** positive rates of BMDMs phagocytizing *Leptospira* were calculated and statistical analyzed by analysis of variance. **(C)** FCM method was employed to confirm the findings. Inactive *Leptospira* was stained by FITC and quenched by trypan blue. **(D)** Macrophages pre-incubated with inhibitors and were co-incubated with the bacteria. Trypan blue was also used to quench the extracellular fluorescence of *Leptospira*. Percentages of phagocytosis with/without adhesion cells (trypan blue not quenched or quenched) were calculated as the number of positive cells with respect to the total events counted. These data were expressed as the mean ± SEM from at least three experiments. **P<0.05*, ***P<0.01*, ****P<0.001*, *ns* no significance.


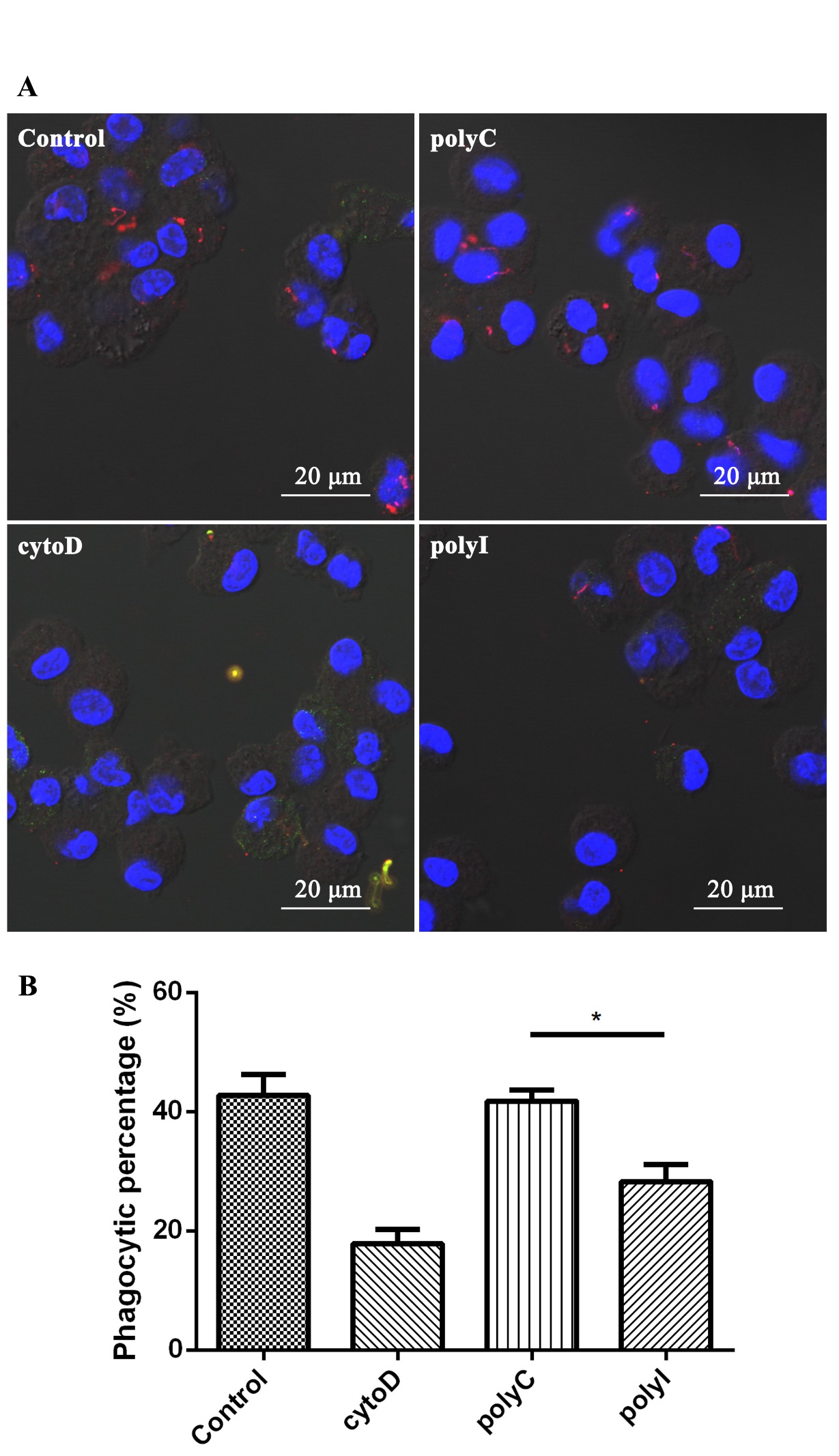


**Supplementary Fig.** **2.** PolyI exhibited the inhibition to the phagocytosis of *L. interrogans* strain 56606v by mouse PMs.

PMs were incubated with active *L. interrogans* strain 56606v in FBS-free medium absence or presence of cytoD (20μM), polyI (100μg/mL) and corresponding concentrations of polyC. Rabbit anti-*L. interrogans* strain 56606v was treated as a specific primary antibody, while FITC-conjugated or TRITC-conjugated anti-rabbit IgG as a secondary antibody were used before and after permeabilization. Confocal microscopic images showed leptospires inside (red) or outside (yellow) of PMs **(A)**. Phagocytic percentages of PMs phagocytizing *L. interrogans* were calculated and statistically analyzed by variance **(B)**. These data were expressed as the mean ± SEM from at least three experiments. **P*<0.05.


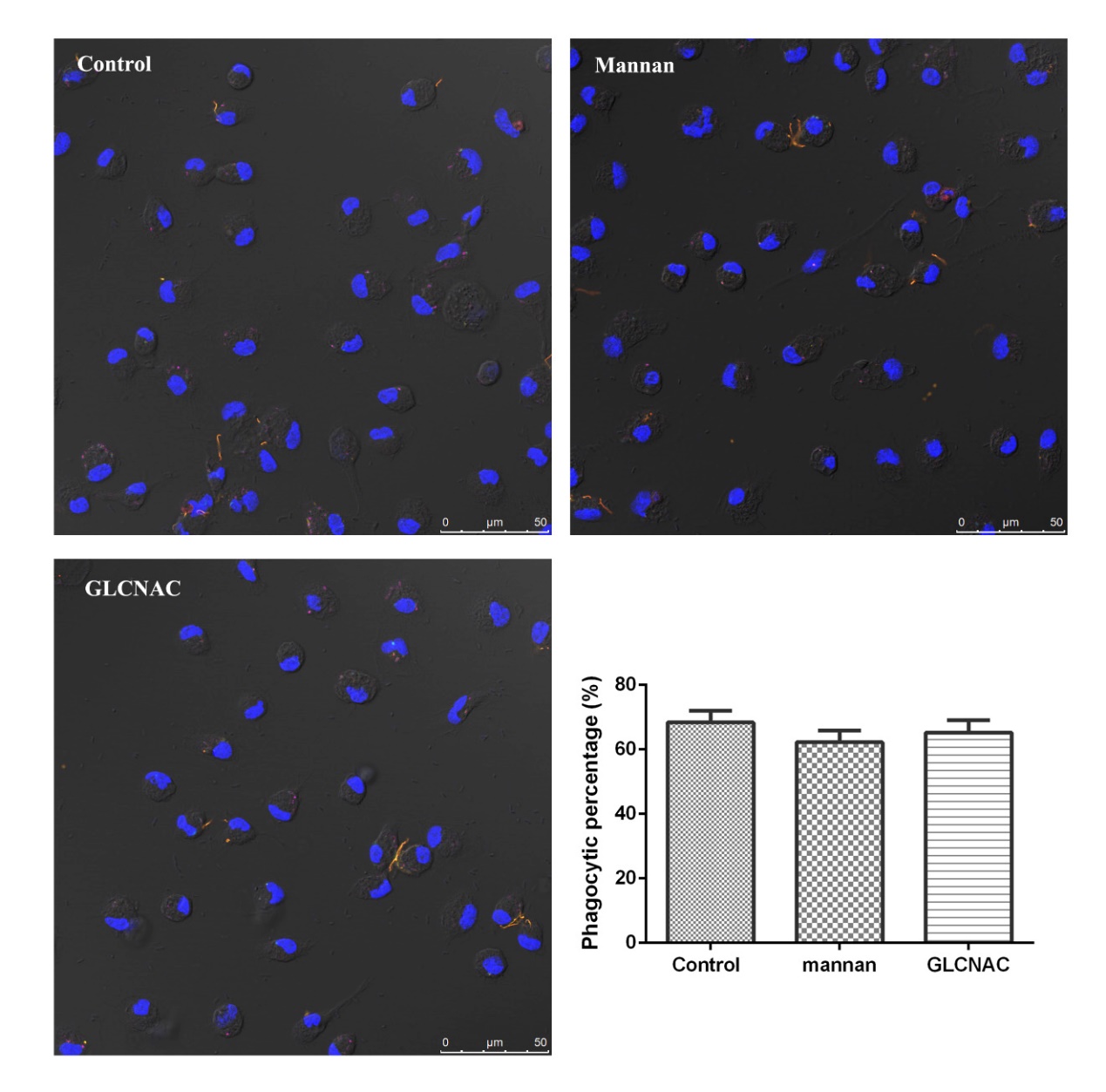


**Supplementary Fig. 3.** Mannan receptor inhibitor exhibited no inhibition to the phagocytosis of *L. interrogans* strain 56606v by mouse PMs.

PMs were incubated with inactive *L. interrogans* strain 56606v in FBS-free medium absence or presence of mannan which was the competitive substance of mannan receptor. Corresponding isotype substance N-acetyl-glucosamine (GLCNAC) was added as controls. Rabbit anti-*L. interrogans* strain 56606v was treated as a specific primary antibody, while FITC-conjugated or TRITC-conjugated anti-rabbit IgG as a secondary antibody were used before and after permeabilization, respectively. After observations by confocal microscopy, positive rates of PMs phagocytizing *Leptospira* were calculated and statistically analyzed. These data were expressed as the mean ± SEM from at least three experiments.


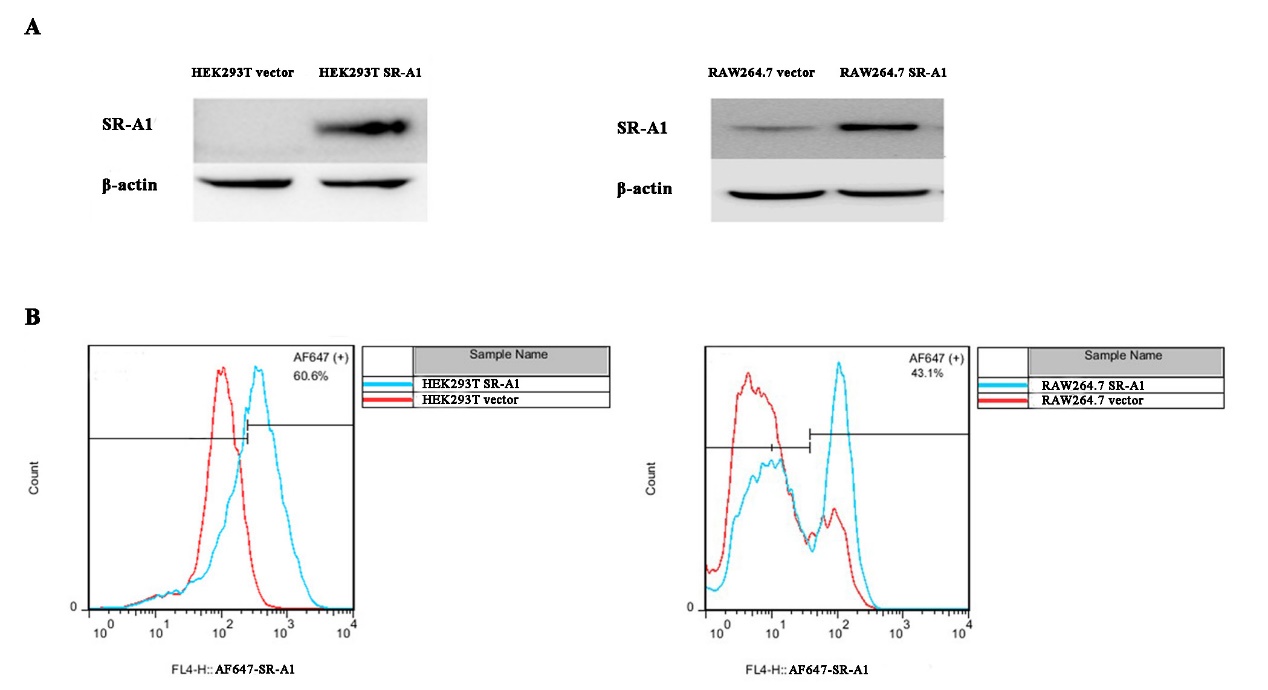


**Supplementary Fig. 4.** Verification of SR-A1 overexpression.

**(A)** SR-A1 overexpression in transfected HEK293T and RAW264.7 cells were detected through Western blot method by SR-A1 primary antibody and HRP-conjugated secondary antibody. Relative protein expression was visualized on the blots. **(B)** Cell surface SR-A1 expression in transfected HEK293T and RAW264.7cells were detected through FCM method by SR-A1 primary antibody and Alexa Flour 647 (AF647) -conjugated secondary antibody. Positive rates of overexpressed cells verifying successful SR-A1 overexpression on cell surface were displayed.


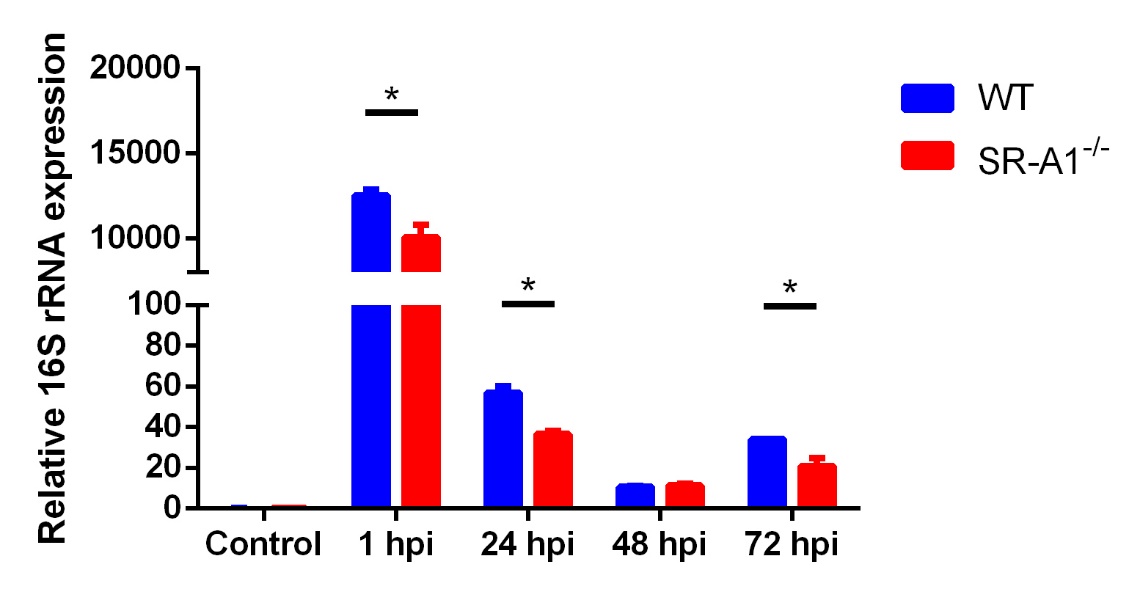


**Supplementary Fig. 5.** Survival of *L. interrogans* strain 56606v in leptospires infected WT and SR-A1^-/-^ PMs.

WT and SR-A1^-/-^ PMs were infected by *L. interrogans* strain 56606v as MOI=100 for 1 h *in vitro*. 100 μg/mL gentamicin was used for 2 h to kill the extracellular leptospires. RNA was extracted from cells of 1 hpi to 72 hpi, and 16S rRNA of leptospires was quantified by real-time PCR. These data were expressed as the mean ± SEM from at least three experiments. **P<0.05*.


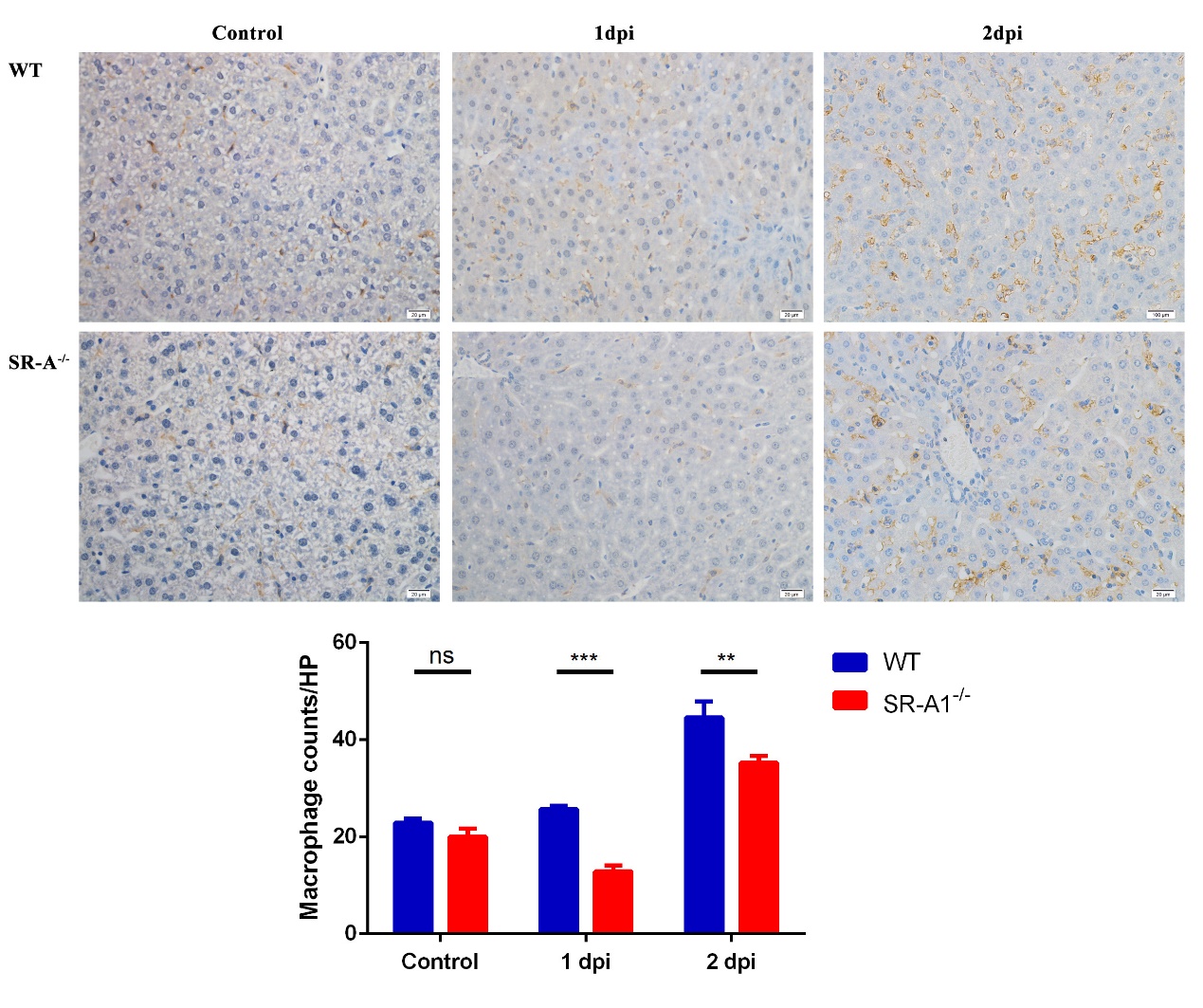


**Supplementary Fig. 6.** Macrophages recruitment in liver was decreased in SR-A1^-/-^ mice after *Leptospira* stimulation *in vivo*.

Livers from WT or SR-A1^-/-^ mice infected with 2×10^8^ leptospires via intraperitoneal route were fixed in neutral buffered 4% formaldehyde, followed by embedding of the tissues in paraffin. Sections were stained for macrophage-specific F4/80 antigen. The average number of F4/80 expressing cells per HP field was calculated. These data were expressed as the mean ± SEM from at least three experiments. ***P<0.01, ***P<0.001, ns* no significance.
